# Incorporating Cryopreservation Evaluations Into the Design of Cell-Based Drug Delivery Systems: An Opinion Paper

**DOI:** 10.3389/fimmu.2022.967731

**Published:** 2022-07-15

**Authors:** Marlene Davis Ekpo, Jingxian Xie, Xiangjian Liu, Raphael Onuku, George Frimpong Boafo, Songwen Tan

**Affiliations:** ^1^ Xiangya School of Pharmaceutical Sciences, Central South University, Changsha, China; ^2^ Department of Pharmaceutical and Medicinal Chemistry, Faculty of Pharmaceutical Sciences, University of Nigeria, Nsukka, Nigeria

**Keywords:** mesenchymal stem cells, cryopreservation, cryoprotectants, chemotherapy, targeted drug delivery

**Graphical Abstract:**
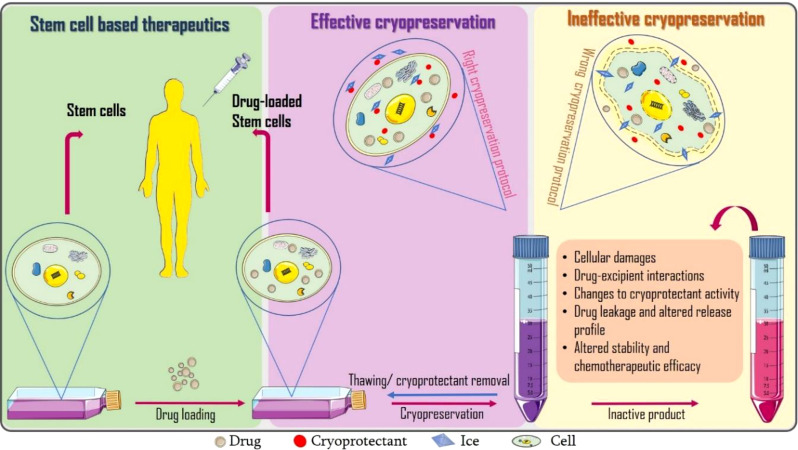
Incorporating cryopreservation evaluations into the design of cell-based drug delivery systems.

## Introduction

Stem cell based therapies capitalize on the desirable features of stem cells with possible modifications to enhance their therapeutic potentials (1). In pharmaceutics and biotechnology, cells have been explored as vehicles for targeted drug delivery (2) especially in cancer treatment because conventional chemotherapy and gene therapy are still limited by factors including poor pharmacokinetics, accumulation in non-cancerous tissues, undesirable side effects and toxicity (3). If these cell-based drug formulations become standardized, cryopreservation is currently the most probable means to ensure long-term storage (4).

Cryopreservation is an indispensable step required for the stability of cell therapies and variables including type and concentration of cryoprotectants, cooling/thawing rate and freezing temperature are critical to ensuring acceptable post-thaw recovery (4). Prior cryopreservation research has revealed that a balance in interactions between ice, water and cryoprotectants is required for optimized cryopreservation outcome. The post-cryopreservation assessments performed involves those to ascertain cell viability, function, and so on (5).

We perceive that drug loading can further alter the dynamics of ice, cryoprotectant and cell interactions resulting to failure of the dosage form. To the best of our knowledge, there are limited studies precisely aimed at assessing the post-thaw stability and therapeutic efficacy of drug loaded (stem) cells. Only few researchers like Lisini et al. (6) have reasoned in this direction. They investigated the post-cryopreservation viability, recovery and release of paclitaxel loaded mesenchymal stem cells after 21 days and suggested further studies should extend this storage time to at least 12 months. Close to evaluating the effects of cryopreservation on drug delivery systems is freeze-thaw stability studies which can provide us with insights on possible deformations to frozen drug delivery systems as presented in **Table 1**.

**Table 1:** Effects of freezing and freeze thaw stability studies of some pharmaceutical formulations.

Formulation	Evaluation	Effect of Cryopreservation/freeze-thaw	REF
Paclitaxel loaded mesenchymal stem cells	Viability, recovery, drug release.	Retained viability and potency of the formulation but loss of cell proliferation and differentiation.	(6)
Hydroxyzine- and cetirizine loaded Multilamellar vesicles (liposomes)	Entrapment efficiency	Percentage entrapment of Hydroxyzine liposomes decreased considerably after 1 month but improved by pH adjustment	(7)
Humanized monoclonal antibody (IgG1)	Freeze-thaw aggregation	Noncovalently linked aggregates composed of native-like monomers were observed after freeze-thaw.	(8)
Recombinant human growth hormone	Safety/immunotoxicity of protein aggregates	Freeze-thaw induced aggregates elicited immunogenicity in mouse model.	(9)
Ethylene glycol/water-based nanofluids containing Al_2_O_3_ nanoparticles	Suspension stability, particle size distribution and thermal conductivity	The assessed parameters were not affected at lower temperature	(10)
Monoclonal Antibody (IgG2)	Fluctuations in buffer pH	Buffer pH increased at below 0° C with sodium phosphate buffer having the greatest change in pH when going from 25 to -30 ° C.	(11)
Insulin loaded PVA hydrogels	Insulin release	Higher freeze-thaw cycles effected insulin release rate and total released amount (from 66 to 38%) negatively.	(12)
Gentamicin palmitate salt and gentamicin sulfate salt loaded bone grafts	Drug release rate and antibiotic activity	Antibiotic activity was not significantly altered after freezing for up to 6 months.	(13)
PLGA microspheres encapsulating FITC-labled dextran	Effect of freezing on sustained release	Freezing increased initial/burst with rapid release kinetic profiles due to high porosity of frozen microspheres.	(14)
Diflunisal loaded chitosan-PVA hydrogels	Swelling capacity, morphology, porosity, drug loading and release profile	Lower freezing temperatures or longer freezing times, resulted in higher porosity and smaller pore sizes and increased intrusion volume. Increasing the number of freezing cycles produced hydrogels with more defined pores and reduced swelling degree.	(15)
Insulin-loaded PLGA nanoparticles	Stability and bioactivity	Co-encapsulation of cryoprotectants alleviated freeze damage and preserved insulin stability and bioactivity.	(16)
Fluconazole-loaded multilamellar liposome	Stability and drug entrapment efficiency	Addition of cryoprotectants (trehalose) before lyophilization produced non-compact and easily reconstituted cakes. Fluconazole entrapment improved significantly (from 63.452% to 91.877%) on addition of trehalose.	(17)

Here in, we opine that research aimed at determining optimal cryogenic storage conditions should be incorporated into the design of cell-based formulations. We discuss some of the parameters that should be evaluated including biocompatibility of cryoprotectants and drugs, cryoprotectant-drug-ice interactions, possible changes to drug entrapment efficacy, drug release, cell viability, and therapeutic efficacy. Suitable molecular dynamics and energy transfer models can be used to decipher the underlying mechanism(s) of drug induced changes in cryopreservation outcomes.

## Stem Cells In Drug Delivery

Conventional chemotherapy are still limited by some factors including poor pharmacokinetics, accumulation in non-cancerous tissues, and toxicity (18). Stem cells are applicable in overcoming these limitations as they can be engineered and used as carriers or vehicles for targeted drug delivery based on the proposition that mesenchymal stem cells (MSCs) undergo chemotaxis toward tumors following the release of chemo-attractants like vascular endothelial growth factor (VEGF) (19). Gao et al. showed that MSCs are promising in this regard as the stem cells carrying paclitaxel loaded nanoparticles (NPs) were able to deliver their therapeutic pay-load to murine orthotopic glioma cells (20). Furthermore, paclitaxel loaded human olfactory bulb neural stem cells (Hu-OBNSCs) and mesenchymal stromal cells have been effective in inhibiting the progression of glioblastoma (20–22). MSCs have also been applied in the delivery of immune biomolecules like cytokines to tumor sites. For instance, transduction of MSCs with an adenoviral expression vector bearing interferon-β gene has been shown to increase the production of interferon-β at the cancer site (23). Similarly, delivery of interleukin expressing MSCs has promoted cytotoxicity to tumor cells via the activation of endogenous natural killer (NK) cells and lymphocytes (24). Extracellular vesicles released by stem cells have also been explored as cancer targeted drug delivery vesicles because of their biocompatibility and minimal immunogenicity. Abnous et al. applied exosomes as carriers in the delivery of doxorubicin to colorectal tumor and results showed that the formulation had better pharmacokinetic properties, tumor location and suppression compared to the free drug (25). These research efforts have shown tremendous potential for clinical application and would be a great drawback if the formulations are rendered inactive by cryopreservation.

## Possible Effects of Cryopreservation And Assessments To Consider In Cryopreservation of Cell-Based Drug Delivery Systems

### Cellular Damage

A growing body of evidence suggests conflicting results regarding the effects of cryopreservation and thawing of stem cells, including extensive physical and biological stress, apoptosis and necrosis, mitochondrial damage, changes in basal respiration and ATP production, damage to cellular structure, telomere shortening and cellular senescence (26), as well as oxidative damages (DNA damage, lipid peroxidation, protein oxidation) from reactive oxygen species released during freezing (27, 28), all of which can inadvertently lead to reduced therapeutic efficacy (29, 30). More details on the principles and protocols for evaluating stem cell viability after cryopreservation can be found in a review by Xie et al. (5). In addition to the potential cryopreservation induced cellular damage (s), we speculate that inclusion of drugs can affect cryopreservation outcome resulting to failure of the dosage form. Lisini et al. reports the loss of proliferation and differentiation in paclitaxel loaded MSCs (6).

### Drug-Excipient Interactions and Changes to Cryoprotectant Activity

The activity of cryoprotectants may be affected by the inclusion of drugs and other excipients. Therefore, the drugs should be analyzed for possible effects on ice (ice recrystallization inhibition (IRI), thermal hysteresis and ice shaping). In fact, a serendipitous discovery of this sort was made by Liu et al. (31) where medium molecular weight sodium hyaluronate (MSH) devoid of prior IRI activity showed significant IRI activity in the presence of red blood cells through an unknown mechanism. The observed IRI activity would imply adjustments to the cryopreservation protocol for red blood cells using MSH. To understand how cryoprotectants, drugs, ice and other cellular components may interact, molecular dynamics modelling studies like ice crystallization (isothermal crystallization) kinetics, and heat and mass transfer modelling (32) can be performed. Furthermore, some cryoprotectants may possess some pharmacological activity that may be incompatible with the loaded drugs or react with drugs to produce toxic effects. Therefore, drug-excipient compatibility studies have to be performed at preformulation to rule out possible chemical or physical interactions that could affect quality, manufacturability and performance of the final product. Other concerns would be if the post-thaw removal of cryoprotectants (especially penetrating cryoprotectants like DMSO and proline) could affect drug entrapment and release properties as discussed in the next section.

### Altered Intracellular Drug Entrapment and Drug Release

Drug release is an important determinant of stability and therapeutic effectiveness of formulations (33). If the drugs leak or the release profile is altered, therapeutic failure is imminent. Other dosage forms can provide cues on how freezing could affect drug entrapment. Kim et al. proposes that the freezing phase of freeze-drying is most probably responsible for the high porosity of polylactic-co-glycolic acid (PLGA) microspheres. Freezing converts the free water molecules present within the pores and their interconnected channels into ice crystals. Following sublimation, the empty cavities spaces retain the shape and dimension of the ice crystal. Cracking (micro-channeling) of the PLGA polymer walls can also arise from overstretching of the pores during ice recrystallization thus further increasing the porosity of the microspheres which in turn is not favorable for sustained or prolonged drug release (14). The application of cryoprotectants in stabilizing lyophilized preparations has been explored in several studies (34–37).

Figueroa-Pizano et al. utilized freezing-thawing in the fabrication of diflunisal loaded chitosan-polyvinyl alcohol (PVA) hydrogels. They discovered that the use of either lower temperatures or prolonged freezing resulted in hydrogels with higher porosity, smaller pores sizes and less swelling capacity, which may not support prolonged drug release (15). Also, freeze-thawing notably reduced the entrapment efficacy of hydroxyzine and cetirizine loaded liposomes (7). With regard to cells, any factor that causes the membrane to lose its integrity would result in drug leakage and distortion of the release profile. These factors include degradation of cell membrane proteins, puncturing of the cell membrane by ice crystals, dehydration, etc. Therefore, cryoprotectants must be carefully screened before selection. Although Lisini et al. reports preserved post-thaw release capacity of paclitaxel based on antitumor potency test (6), it would still be necessary to quantify the drug released at each time for a better understanding of the drug release kinetics and also compare the drug entrapment before and after cryopreservation.

### Altered Stability and Chemotherapeutic Efficacy

Stability and therapeutic efficacy are paramount parameters to be assessed during formulation studies. But presently, there is a gap in literature to address the post-cryopreservation performance of cell-based formulations. Lisini et al. reports that efficacy of paclitaxel loaded stem cells was not significantly altered by cryopreservation (6). Fonte et al. assessed the effect of freezing on the structural stability of encapsulated insulin prior to lyophilization (16). Their results showed pH fluctuations caused by temperature-related changes to crystallization, pKas, solubility, eutectics, and cryoconcentration (11). Coraça-Huber et al. examines the post-thaw antibiotic activity of gentamicin loaded bone grafts following cryopreservation at -80°C for up to six (6) months. Results showed uncompromised antibiotic activity of the formulations against *Staphylococcus aureus* (13). Freezing and thawing can induce damage of protein therapeutics (antibodies, lipids, proteins, DNA, genes) resulting in depletion or total loss of therapeutic activity. Protein aggregation can be as a result of altered conformational stability at water–ice interface or cryoconcentration of solutes and proteins in the liquid phase (38). Cryoconcentration frequently occurs during slow freezing where various peptide components and other solutes separate from the water–ice interface creating a concentration gradient proximal to the ice front (39). The resultant phase separation and probable loss of pH buffering are key factors responsible for protein aggregation and structural damage. The concentrated solutes can also contribute to aggregation by disturbances to protein thermodynamic stability. On the other hand, less water recrystallization occurs during rapid freezing leading to the formation of smaller ice crystals causing exposure of proteins and other solutes to a greater water-ice crystal interface where they can be adsorbed and concentrated leading to increased aggregation, loss of structural integrity and therapeutic efficacy. Freeze-thaw induced protein aggregation and perturbed therapeutic activity of a model monoclonal antibody (mAb) therapeutic- Trastuzumab can be found in a study by Dash and coworkers (40). Hence, freezing protocols must be designed and optimized to favor the cryopreserved material (41). Horn et al. confirms the effectiveness of cryoprotectants for maintaining stability of immunoglobulin (IGG) solutions. They discovered significantly higher formation of monomer aggregates IGG formulations without cryoprotectants (42). Similarly, Jain et al. reports significant reduction in freeze-thaw damage of mAb-1 attained by prior optimization of cryopreservation protocols during pilot scale studies (43). Furthermore, Liu and co-authors suggest that freezing induced disturbances to the tertiary structure of mAbs are reversible or irreversible depending on the pH of the system or the type of excipients included in the formulation (44). Therefore, thorough research has to be conducted before selecting excipients (cryoprotectants and buffers) for the formulation and cryopreservation of cell-based drug delivery systems.

## Conclusion

Based on the complexity of novel cell-based drug delivery systems, it would be detrimental to assume the absence of alterations to the formulation after cryopreservation. To achieve the desired therapeutic effect, an equilibrium must be maintained between the several factors that could influence product stability. These factors should include but are not limited to cryoprotectant type and concentration, drug type and concentration, cell type, freeze/thaw rate, mode of freezing. The optimal conditions for storage and transportation can only be determined through scalable and reproducible research. As observed from lisini et al., the paclitaxel loaded stem cells possessed appreciable chemotherapeutic activity after cryopreservation with 10% dimethyl sulfoxide (DMSO) supplemented with sodium chloride (0.9%w/v) and 5% human albumin but these results cannot be extrapolated to other cell types, cryoprotectants, drugs and storage duration. In fact, there is a growing need to find better substitutes to DMSO for cryopreservation owing to its unwanted effect on cryopreserved material and in humans after clinical application. Hopefully, future research in this direction will serve as guide to perform similar studies for other drug delivery systems that requires freezing or cryopreservation as an indispensable technique during fabrication, storage and transportation. Future studies can also investigate cryoprotectants with drug carrier properties e.g., graphene oxide, nanocellulose and PVA. If this is achievable the post-thaw removal of such cryoprotectants before clinical application would be eliminated.

## Author Contributions

Conceptualization, ME, ST. Writing—original draft preparation, ME, JX, RO. Writing—review and editing, ME, XL, GB, and ST. Supervision and approval, ST. All authors contributed to the article and approved the submitted version.

## Conflict of Interest

The authors declare that the research was conducted in the absence of any commercial or financial relationships that could be construed as a potential conflict of interest.

## Publisher’s Note

All claims expressed in this article are solely those of the authors and do not necessarily represent those of their affiliated organizations, or those of the publisher, the editors and the reviewers. Any product that may be evaluated in this article, or claim that may be made by its manufacturer, is not guaranteed or endorsed by the publisher.
