# Erratum: Incorporating cryopreservation evaluations into the design of cell-based drug delivery dystems: An opinion paper

**DOI:** 10.3389/fimmu.2022.1020829

**Published:** 2022-08-31

**Authors:** Frontiers Production Office

**Affiliations:** Frontiers Media SA, Lausanne, Switzerland

**Keywords:** mesenchymal stem cells, cryopreservation, cryoprotectants, chemotherapy, targeted drug delivery

Due to a production error, there was a mistake in affiliation two of this article. Instead of “School of Pharmacy, Taipei Medical University, Taipei, Taiwan”, it should be “Department of Pharmaceutical and Medicinal Chemistry, Faculty of Pharmaceutical Sciences, University of Nigeria, Nsukka, Nigeria”.

The publisher sincerely apologizes for this mistake.

The original version of this article has been updated.

